# B-mode ultrasonography and ARFI elastography of articular and peri-articular structures of the hip joint in non-dysplastic and dysplastic dogs as confirmed by radiographic examination

**DOI:** 10.1186/s12917-023-03753-7

**Published:** 2023-10-02

**Authors:** Rafael Kretzer Carneiro, Igor Cezar Kniphoff da Cruz, Beatriz Gasser, Bruna Lima, Luiz Paulo Nogueira Aires, Márcio Poletto Ferreira, Ricardo Andres Ramirez Uscategui, Robson Fortes Giglio, Bruno Watanabe Minto, Marcus Antônio Rossi Feliciano

**Affiliations:** 1 São Paulo State University “Júlio de Mesquita Filho”, UNESP, Jaboticabal, SP Brazil; 2 State University of Santa Catarina, UDESC, Lages, SC Brazil; 3 Federal University of Santa Maria, UFSM, Santa Maria, RS Brazil; 4 Federal University of the Jequitinhonha and Mucuri Valleys, UFVJM, Unaí, MG Brazil; 5 Federal University of Rio Grande do Sul, UFRGS, Porto Alegre, RS Brazil; 6 University of Tolima, UT, Ibagué, Colombia; 7 University of Georgia, UGA, Athenas, GA USA; 8 University of São Paulo, USP, Pirassununga, SP Brazil

**Keywords:** Canine, Canine hip dysplasia, Hip laxity, Ultrasonography, Acoustic radiation force impulse

## Abstract

**Background:**

Canine hip dysplasia is a common orthopedic disease in veterinary practice. The diagnosis is made by radiographic examinations that evaluate bone alterations associated with hip dysplasia. Although radiographic examination is the gold standard for diagnosis, it does not allow a detailed evaluation of soft tissues such as the joint capsule and periarticular muscles. This study aimed to evaluate the accuracy of B-mode ultrasonography and acoustic radiation force impulse (ARFI) elastography in assessing the joint capsule and periarticular muscles of dogs using the Orthopedic Foundation of Animals (OFA) classification and the distraction index (DI) in the early and late diagnosis of hip dysplasia. This study sought to propose a protocol for the ultrasonographic evaluation of the structures involved in canine hip dysplasia.

**Methods:**

Radiographic and ultrasonographic evaluations were performed on 108 hip joints of 54 dogs. Thirty dogs were older than 2 years and 24 were aged between 4 and 10 months.

**Results:**

It was verified that an increase in pectineus muscle stiffness (cutoff value > 2.77 m/s) by elastography in some dysplastic dogs and an increase in the thickness of the joint capsule (cutoff value > 0.9 mm) in B-mode ultrasonography, were associated with a distraction index > 0.5, with both having a positive correlation. In B-mode ultrasonographic evaluation, the presence of signs of degenerative joint disease, such as irregularities of the cranial edge of the acetabulum and femoral head, were associated with a distraction index > 0.5 in canines, with a specificity of 94%. In adult dogs, the findings of degenerative joint disease on ultrasound were associated with a diseased OFA classification (P < 0.05). Measurement of the joint capsule > 1.10 mm was diagnostic for dysplasia in unhealthy dogs by OFA.

**Conclusions:**

ARFI elastography has shown that the pectineus muscle may experience changes in stiffness in dysplastic animals. Additionally, changes in joint capsule thickness can be identified in B-mode in young and adult dogs with dysplastic joints, which contributes to the diagnosis of hip dysplasia.

**Supplementary Information:**

The online version contains supplementary material available at 10.1186/s12917-023-03753-7.

## Background

Canine hip dysplasia (CHD) is a progressive disease characterized by joint instability caused by the malformation of the hip joint [1]. It occurs under the influence of hereditary and environmental factors [2, 3] and is more prevalent in medium- and large-sized dog breeds [4, 5]. Affected animals may show clinical signs, such as lameness of the hind limbs, exercise intolerance, bunny hopping gait, and muscular atrophy [6].

Radiographic examination of the hip is the gold standard for diagnosing dysplasia [5, 7, 8]. Numerous radiographic approaches can be employed to assist in the investigation of secondary disease [9]. Extended hip projections are used by the Orthopedic Foundation for Animals (OFA) to assess bone changes associated with hip dysplasia in dogs ≥ 24 months of age [9, 10]. Currently, phenotypic screenings focus on an alternative methodology for joint laxity [11]. Among them, the PennHip, developed at the University of Pennsylvania, stands out as an accepted and validated approach [12] for immature animals aged 16 weeks or more that evaluates the index of joint distraction [13, 14].

For hip movement to occur, periarticular muscles must be activated to facilitate joint stability [15]; however, the role of periarticular muscles in the pathogenesis of hip dysplasia is still poorly understood. We hypothesize that in animals with hip dysplasia it is possible to verify the increase in tissue resistance of the main muscles responsible for hip mobility. In medicine, ultrasound evaluation of the hip has been used since 1980, when it was first developed by Graf with the objective of identifying instabilities in the hip joints of newborn children [16]. In veterinary medicine, a similar technique has been described in dogs, but so far the results have been inconsistent with the development of dysplasia in adult dogs [17]. We believe that the anatomical conformation and biomechanics of the hips in dogs is a fundamental factor for the divergence of results. In addition, the angles established for the evaluation may be different for each breed, and breed standardization is important to better direct the ultrasound measurements in this technique.

Acoustic radiation force impulse (ARFI) elastography is an ultrasonographic technique that allows quantitative and qualitative measurement of changes in tissue stiffness [18–20] by evaluating deformation according to Hooke’s law. In general, elastography can be used to differentiate affected tissue (with morphostructural alterations) from normal tissue [21]. This technique has been explored for several clinical purposes and introduced into medical diagnostic routines for specific uses, such as the evaluation of liver transplantation in humans [22], splenic alterations [20], and characterization of mammary lesions in dogs [23, 24].

Regarding the changes correlated with hip dysplasia, few studies have demonstrated the applicability of ARFI elastography as a diagnostic aid. In a preliminary study comparing the pectineus muscles of non-dysplastic and dysplastic dogs, it was found that dysplastic dogs have greater pectineus muscle stiffness (3.49 ± 1.00 m/s) than non-dysplastic dogs (2.41 ± 0.59 m/s), demonstrating that there is an overload on the inner muscles of the hip, making it less elastic in dysplastic dogs [25].

Considering the possibility of an early diagnosis of CHD with the aid of ultrasonography, this study aimed to evaluate the joint capsule and periarticular muscles of the hip in dysplastic and non-dysplastic dogs using the ARFI elastography technique and B-mode ultrasonography by determining the predictive values of changes in tissue elasticity and thickness of the joint capsule. We correlated these evaluations with radiographic findings of the hip assessed through the distraction index (DI) and the Orthopedic Foundation of Animals (OFA) classification, in order to determine the applicability of ultrasound methods in the diagnosis and screening of hip dysplasia.

## Results

A total of 108 hip joints of 54 dogs were evaluated. Dogs were separated into groups of young and adult animals. Twenty-four dogs aged between 4 and 10 months were included in the group of young animals. The average age of these animals was 7.29 months ± 2.18 with a mean weight of 20.08 ± 6.16 kg. Thirty dogs aged ≥ 2 years were included in the group of adult animals. In this group, the average age was 5.38 years ± 2.99 with an average weight of 34.31 ± 7.05 kg.

### Radiographic findings - distraction index (DI)

The distraction index was used only in the group of young dogs. DI was the gold standard for assessing joint laxity. Seventeen joints were graded as non-dysplastic (DI ≤ 0.5) with a mean of 0.42 ± 0.12, while 31 joints were scored as dysplastic (DI > 0.5), with a mean of 0.84 ± 0.17. Patients with a DI ≤ 0.5 had a mean age of 7.41 months ± 2.43 and a mean weight of 17.49 kg ± 3.76. Patients with a DI > 0.5 had a mean age of 7.23 months ± 2.03 and a mean weight of 21.49 kg ± 6.70.

### Radiographic findings – orthopedic foundation for animals (OFA)

The OFA assessment was used only in the group of adult dogs. The OFA assessment criteria were used as the gold standard for determining whether a dog’s joint was non-dysplastic or dysplastic due to changes in degenerative joint disease secondary to hip dysplasia.

Twenty-nine joints were considered non-dysplastic (≤ 3), and 28 were classified as dysplastic (≥ 5) based on the agreement of at least two raters. Three joints were discarded because there was no agreement between the non-dysplastic and dysplastic classifications by at least two radiologists and these joints were not reassessed after 6 months and were not included in the assessment. The animals that were scored as non-dysplastic and dysplastic by two or three evaluators had an average grade to guide the statistical tests.

Non-dysplastic dogs had a mean age of 5.17 ± 3.45 years and a mean weight of 34.65 ± 6.97 kg. The dysplastic dogs had a mean age of 5.54 ± 2.39 years and a mean weight of 35.15 ± 5.64 kg.

### B-mode ultrasound

**Fig. 1:**
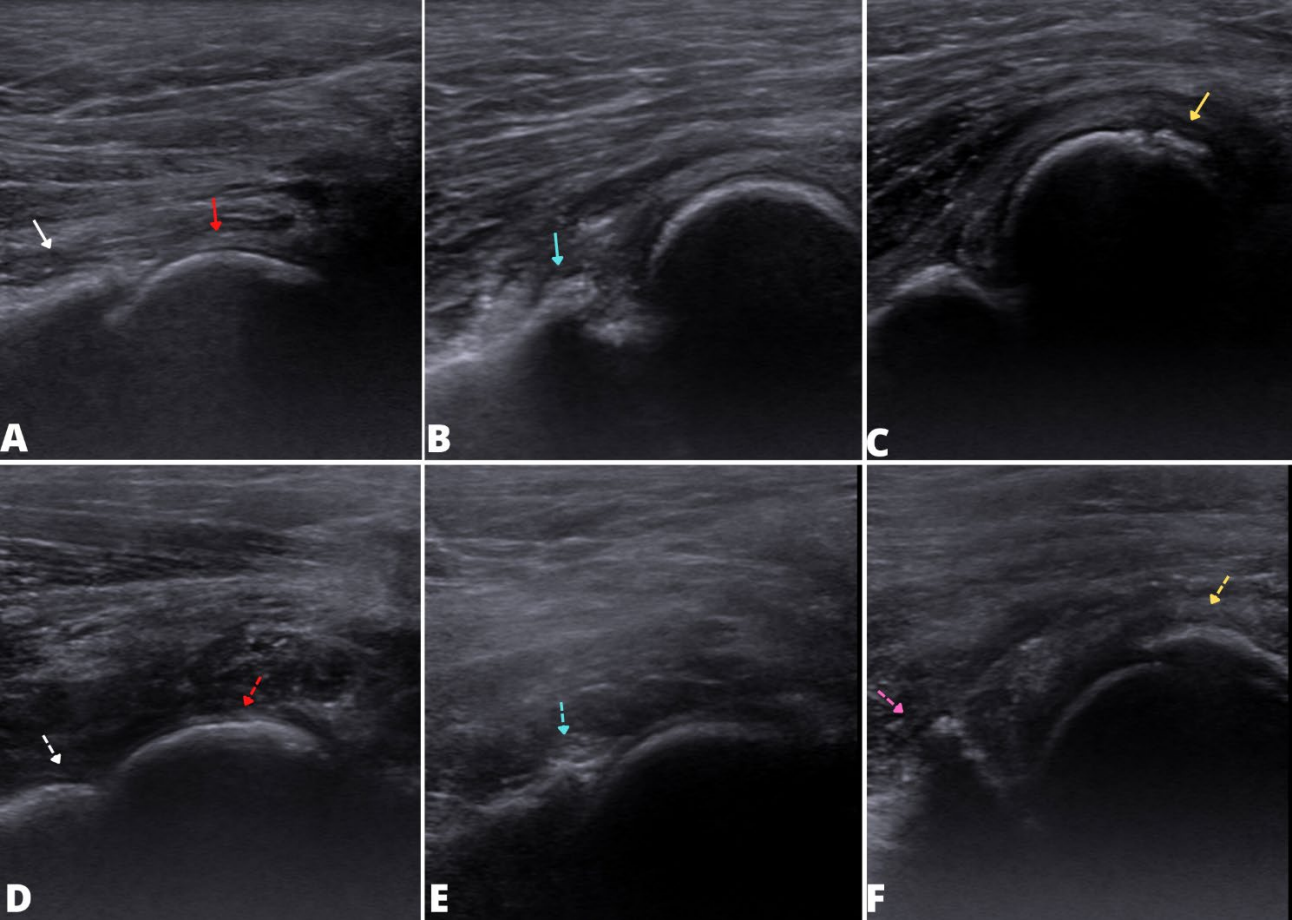
B-mode ultrasonography images of the hip joint in puppies (between 4 and 10 months) (**a**, **b** and **c**) and adult (≥ 2 years) (**d**, **e** and **f**) canine patients. Note the absence of ultrasonographic changes in (**a**) and (**d**) the acetabular cranial rim (white arrow and dotted white arrow) and femoral head (red arrow and dotted red arrow). In (**b**), (**e**) and (**f**) bone irregularities are present on the cranial edge of the acetabulum (blue arrow, dotted blue arrow and dotted pink arrow). Images in (**c**) and (**f**) demonstrate an irregularity in the femoral head (yellow arrow)

Ultrasound examination was performed on all dogs. In the group of young animals, bone irregularity of the cranial acetabular rim and femoral head were associated with joints with a distraction index > 0.5 (P = 0.009). All joints with sonographic changes (n = 10) were radiographically classified as diseased (DI > 0.5) (Fig. 1). In adult animals, bone irregularities were also associated with diseased hips classified by OFA (P = 0.001) (Fig. 1). The mean, standard deviation, prevalence, sensitivity, specificity, positive predictive values, and negative predictive values are all shown in Table 1.

**Table 1:** Findings of bone alterations by B-mode ultrasonography compared to DI in dogs between four and 10 months old (puppies) and animals older than 2 years old (adults)

Evaluation	Nº. Healthy Hips	Nº Sick Hips	mean ± SP Healthy	mean ± SP Sick	95% CI Healthy	95% CI Sick	P-value	Prev	Se (%)	Sp (%)	PPV (%)	NVP (%)
Puppy dogs	38	10	0.64 ± 0.24	0.87 ± 0.19	0.56–0.72	0.72–1.03	0.009	65	29	94	90	42
Adult dogs	37	20	3.52 ± 1.54	5.19 ± 1.82	2.98–4.07	4.45–5.92	0.001	52	45	79	70	57

DI – distraction index; Nº. - number; SD - standard deviation; CI - confidence interval; Prev - prevalence; Se - sensitivity; SP - specificity; PPV - positive predictive value; NPV - negative predictive value.

**Fig. 2:**
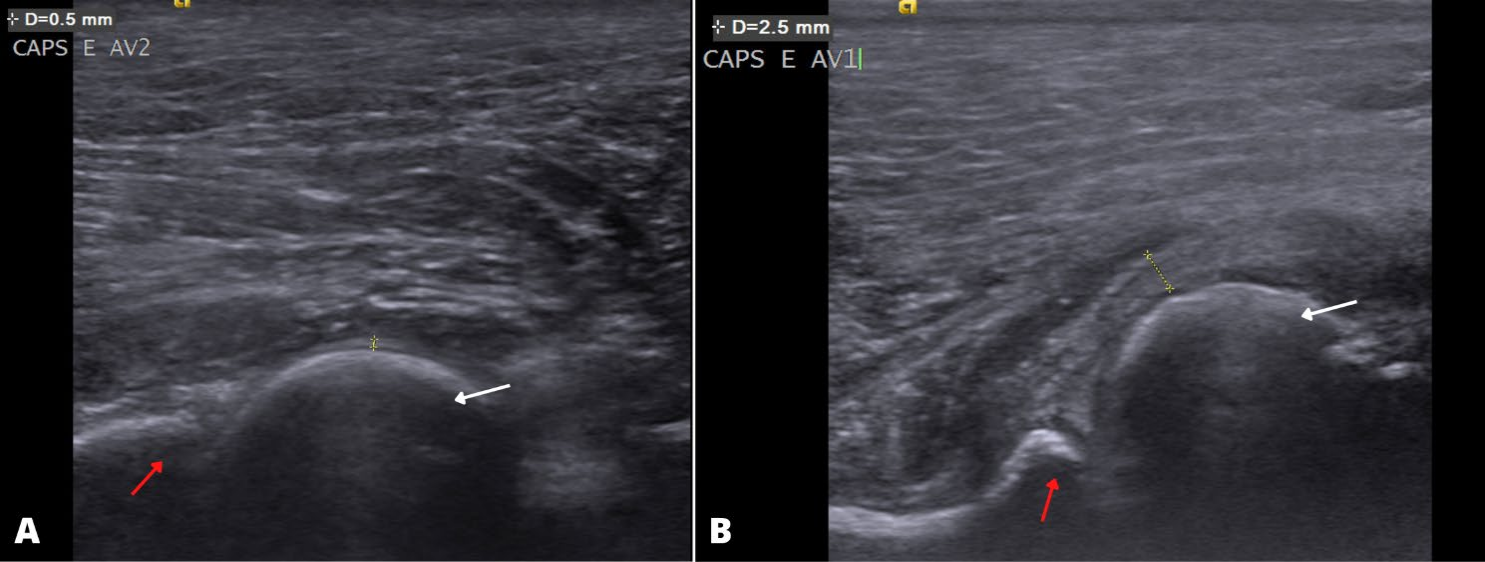
B-mode ultrasonography images of the measurement of the joint capsule of the hip joint of a (**a**) non-dysplastic (**b**) and dysplastic puppy. In (**a**) and (**b**), note the femoral head (white arrow), the cranial edge of the acetabulum (red arrow), and the measured joint capsule (yellow line). Note in (**a**) the capsule of a non-dysplastic joint (DI ≤ 0.5) measuring 0.5 mm and in (**b**) the capsule of a diseased joint (DI > 0.5) measuring 2.5 mm

Measuring the thickness of the joint capsule in young animals was a diagnostic feature (Fig. 2) (P < 0.0001), with a cut-off point > 0.9 mm as an indication of joint laxity (DI > 0.5) showing 70.6% sensitivity, 83.9% specificity, an area under the curve (AUC) of 85.2%, and an odds ratio (OR) of 4.6 with both correlated variables showing a positive correlation (0.79) (Table 2).

**Table 2:** Results of joint capsule thickness by B-mode ultrasonography and ARFI elastography of hip periarticular structures compared to distraction index (DI) in puppies aged between four and 10 months

Structures	Thic. (mm) mean ± DP (DI ≤ 0.5)	Thic. (mm) mean ± DP (DI > 0.5)	SWV (m/s) mean ± DP (DI ≤ 0.5)	SWV (m/s) mean ± DP (DI > 0.5)	P-value	P- diagnosis	Cutoff (mm)	Se (%)	Sp (%)	AUC (%)	Odds Ratio	Cor.
Capsule Thickness	0.89 ± 0.57	1.70 ± 0.75			0	< 0.0001	> 0.9	70.6	83.90	85.2	4.6	0.79
Caspule			5.05 ± 0.79	4.99 ± 0.77	0.82							
Gluteus medius			2.60 ± 0.53	2.51 ± 0.60	0.61							
Vastus lateralis			3.42 ± 0.50	3.38 ± 0.66	0.84							
Pectineus			3.01 ± 0.70	3.65 ± 1.37	0.04	0.20	> 2.77	41.2	77.4	61.2	1.82	0.32
Gracile			2.48 ± 0.42	2.54 ± 0.47	0.68							
Adductor			1.93 ± 0.33	1.92 ± 0.36	0.89							
Rectus femoris			2.58 ± 0.47	2.43 ± 0.43	0.30							

Thic - thickness; SWV - shear wave velocity; SD - Standard deviation; DI - distraction index; Se - sensitivity; Sp - specificity; AUC - area under the curve; Cor - correlation

In adult animals as well as in puppies, measuring capsular thickness simplifies the diagnosis into non-dysplastic and dysplastic animals when compared to the OFA classification that uses a cutoff point of > 1.10 mm as an indication of dysplastic animals. Furthermore, a positive correlation exists between these two variables (0.63). The sensitivity, specificity, AUC, and OR values are shown in Table 3.

**Table 3:** Results of joint capsule thickness by B-mode ultrasonography and ARFI elastography of hip periarticular structures compared to OFA grading in adult dogs aged ≥ 2 years

Structures	Thic. (mm) mean ± SD (Healthy)	Thic. (mm) mean ± SD (Sick)	SWV (m/s) mean ± SD (Healthy)	SWV (m/s) mean ± SD (Sick)	P-value	P-diagnosis	Cutoff (mm)	Se (%)	Sp (%)	AUC (%)	Odds Ratio	Cor
Capsule Thickness	1,05 ± 0,54	2,05 ± 1,24			0	< 0,0002	> 1,10	72,4	78,57	78,26	3,21	0,63
Caspule			4,99 ± 1,15	5,14 ± 1,36	0,65							
Gluteus medius			2,47 ± 0,48	2,42 ± 0,79	0,77							
Vastus lateralis			3,26 ± 0,66	3,13 ± 0,76	0,48							
Pectineus			3,24 ± 0,52	3,14 ± 0,70	0,57							
Gracile			2,49 ± 0,50	2,53 ± 0,46	0,73							
Adductor			2,08 ± 0,39	2,13 ± 0,44	0,65							
Rectus femoris			2,50 ± 0,33	2,52 ± 0,34	0,82							

Thic - thickness; SWV - shear wave velocity; SD - Standard deviation; Se - sensitivity; Sp - specificity; AUC - area under the curve; Cor - correlation.

**Fig. 3:**
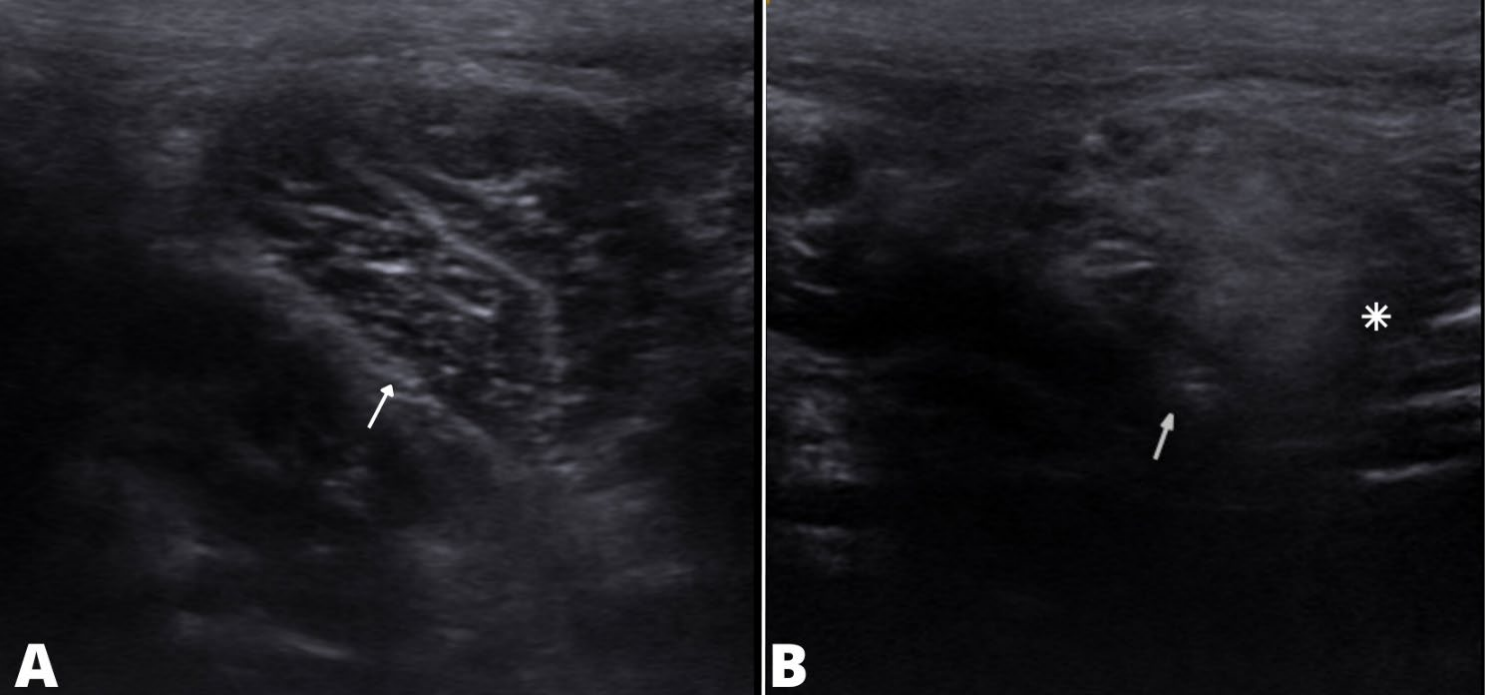
B-mode ultrasonographic evaluation of the pectineus muscle of a dog (**a**) without areas of muscle contracture (**b**) and a dog with areas of muscle contracture. In (**a**) and (**b**) the pectineus muscle can be seen (white arrow). In (**b**) a hyperechogenic area (asterisk) is observed with fascicular and perimysial pattern loss. This was not observed in the structure in (**a**)

During the evaluation of echotexture and echogenicity of the muscle structures, no changes were found in Group 1. Furthermore, only the pectineus muscle demonstrated hyperechogenic areas with changes in the echotexture of the muscle fascicles and changes in the perimysium in the five joints of adult patients who were classified as dysplastic patients by OFA (Fig. 3).

### ARFI elastography

ARFI elastography was performed on all dogs. In the quantitative elastographic study, greater stiffness was observed only in the pectineus muscle of young animals (P = 0.048) joints with a DI > 0.5 (3.65 ± 1.37 m/s) in relation to the hips with a DI ≤ 0.5 (3.01 ± 0.70 m/s). However, this was not indicative of hip dysplasia (P = 0.20). No associations were observed between the shear velocity of the other muscle structures and the joint capsule with DI on elastography (P > 0.05) (Table 2).

In adult animals (≥ 2 years old), no statistical difference was found between ARFI elastography and the periarticular structures of non-dysplastic and dysplastic dogs (Table 3).

## Discussion

The present study provides unprecedented information regarding sonographic findings of the articular and periarticular structures of the hip joint in canine patients with and without CHD evaluated using the joint distraction method and OFA. In addition, the established ultrasound evaluation protocol may contribute to the investigation of dysplasia in puppies, between 4 months to 10 months old, and adults due to the diagnostic characteristics established by changes in joint capsule thickness.

Although the gold standard of CHD diagnosis is radiographic examination [8, 10], the results of this study demonstrated that ultrasonographic evaluation can be used as a screening method and diagnostic aid for CHD and that the association of both imaging methods can bring greater accuracy in disease investigation.

In medicine, ultrasound of the hip joint is utilized to guide the diagnosis of developmental dysplasia of the hip (DDH) in newborns using the Graff methodology [26]. Fischer et al. [17] attempted to apply this methodology in dogs under 50 days old but were unable to obtain a correlation with dysplasia. There is a lack of studies providing diagnostic information for CHD in puppies and adults using the ultrasound approach. This study found that capsule thickness was associated with diseased animals, allowing for determination of its diagnostic value when correlated with radiographic evaluation by DI and OFA in dogs between 4 and 10 months of age and adults (≥ 2 years), respectively. This association can be explained by the fact that CHD generates joint laxity [3], which intensifies the stress suffered by the joint capsule over time and is compatible with the degeneration of joint structures. We suggest conducting new ultrasound studies with newborn dogs that prospectively study the joint capsule to better identify early alterations in soft tissues that may be correlated with CHD.

A report suggested that capsule thickening is progressive, as inflammatory factors are still present in the diseased joint [27], which contributes to changes in joint structures. Furthermore, the cut-off point for young dogs was lower than that for adults, with a positive correlation between the variables.

The OFA grading method may underestimate osteoarthritis in adult dogs compared to the distraction index method [28]. It is important to interpret capsule findings and bone alterations because these structural changes may be correlated with animals having less joint laxity in adulthood, which highlights the usefulness of ultrasound diagnosis by the distraction index. Therefore, we believe that ultrasonography can be used as an auxiliary method in diagnosing hip dysplasia, especially in borderline cases or when there is disagreement among raters using the OFA scale.

In our study, three joints were discarded and not reassessed after six months for definitive diagnosis of CHD by OFA. However, using the capsule thickness, the joints could be classified as dysplastic or non-dysplastic, eliminating the need for new radiographic examinations, reducing radiation exposure and anesthetic risk to patients. By considering the capsular biological changes in the face of dysplasia, veterinarians can associate imaging methods in borderline animals, providing greater certainty to the diagnosis and decision-making in the reproductive management.

Although a statistical analysis of the echotexture and echogenicity evaluations was not carried out due to the subjectivity of the evaluation and the limited sample size, the ultrasonographic evaluation of the pectineus muscle is important for the therapeutic approach, as there is a positive correlation between the presence of muscular fibrosis and hip dysplasia [29]. This muscle plays a fundamental role in the biomechanics of the hip [15], justifying the change in the pattern of the identified fibers seen in B mode ultrasound in some patients in this study.

ARFI elastography has provided important information about the morphostructural behavior of tissues, making it possible to differentiate healthy structures from diseased ones in dogs through variations in tissue stiffness [18–20]. Although this study failed to obtain diagnostic results with elastography for CHD in joint and periarticular structures, it was possible to identify changes in the elasticity of the pectineus muscle between non-dysplastic and dysplastic young animals. The change in the rigidity of the pectineus in dogs has already been correlated with dysplasia in adult dogs [25], but based on our findings, we believe that the pectineus is not the main structure of the hip that is compromised by CHD, but rather the joint capsule, as it is the main structure responsible for the stabilization and movement of the hip joint [30].

In this study, no difference was found in the elasticity of the joint capsule using the ARFI method between non-dysplastic and dysplastic animals. The authors believe that this may be related to the size of the caliper used to obtain the shear wave velocity (SWV) results, since the capsule is a small structure, and the area of interest may have approached structures adjacent to the capsular evaluation, overestimating or underestimating the results. Therefore, histopathological studies of the joint capsule should be encouraged to assess structural changes in dysplastic dogs, in order to provide more concrete information about the interference of CHD in the capsular structure.

In this study, we demonstrated the importance of evaluating the articular and periarticular structures in dogs, proving that ultrasonography can help in the screening and diagnosis of CHD and allowing for the prompt treatment of dysplastic animals. In addition, ultrasound examination is a painless, non-invasive procedure that does not require sedation, which provides greater safety to patients.

This study has limitations, among which is the difficulty we encountered in performing homogeneous sampling (weight, sex, breed, and age) between the groups, which could have changed the characteristics of the joint and periarticular structures and may have interfered with the elastography values. In addition, some elastography values may have overlapped, such as the pectineus elasticity findings in young animals, which may be related to the study’s inability to diagnose changes in tissue elasticity using the ARFI method in patients with hip dysplasia.

## Conclusions

ARFI elastography has shown that the pectineus muscle may experience changes in stiffness in dysplastic animals. Additionally, changes in joint capsule thickness can be identified in B-mode in young and adult dogs with dysplastic joints, which contributes to the diagnosis of hip dysplasia.

## Methods

### Experimental design and animals

This study was carried out according to the ARRIVE guidelines 2.0 (2020). Data were collected prospectively between August 2020 and March 2022. Dogs of different breeds and ages from the hospital routine, with or without a history of hip dysplasia, were included. The animals were divided into two groups: Group 1 consisted of young dogs aged between 4 and 10 months weighing more than 10 kg, and Group 2 consisted of adult animals aged ≥ 2 years and weighing more than 15 kg. All patients were previously evaluated by the small animal orthopedic service available in the veterinary hospital of the institution, in order to exclude animals with other orthopedic morbidities from the evaluation.

### Radiographic examination

All dogs were clinically evaluated and blood samples were collected for laboratorial evaluation (complete blood count, albumin, creatinine, and alkaline phosphatase) before the radiographic study. Patients with changes in clinical or laboratory test results were excluded from the evaluation. All dogs were sedated for radiographic examination. In both groups, the animals received acepromazine (0.02 mg/kg), butorphanol (0.3 mg/kg) and midazolam (0.2 mg/kg) intramuscularly. After ten minutes, venoclysis was performed with a 22 g catheter and, when necessary, an intravenous bolus of propofol was administered. Throughout the anesthetic procedure, the animals were monitored with pulse oximetry and vascular Doppler and received 100% oxygen via a face mask.

Radiographic examinations were performed with a conventional radiography device (RG150/100gl, Siemens®, Munich, Germany). Images were obtained in a cassette digitalization (imaging plate, AGFA-Gevaert N.V., Morstel, Belgium) measuring 35 × 43 cm and then scanned by a computerized system into a digital format (model CR-30, AGFA-Gevaert N.V, Mortsel, Belgium) and evaluated with a workstation equipped with a specific Digital Imaging and Communications in Medicine (DICOM) software. The patients were positioned by the same operator, with the aim of evaluating the hip joints.

Three radiographic projections were obtained in young dogs: ventrodorsal, distraction, and joint compression. In adult dogs, only the ventrodorsal projection was obtained.

To perform the ventrodorsal radiographic projection, the dogs were positioned in dorsal recumbency on the radiography table, with the pelvic limbs fully extended, femurs parallel, and medially rotated with the patella centralized in the trochlea [31].

To perform the distraction and compression radiographic the dogs were positioned in dorsal recumbency using a distractor, with parallel bars positioned between the proximal femurs, and the limbs positioned with the hip and stifle joints at 90º. An adduction force was applied to the stifle by the manipulator to obtain separation between the femoral head and acetabulum [32]. Neutral compression projection was obtained with abduction of the pelvic limbs followed by slight internal compression in the stifle joints to reorient the femoral head in the acetabulum with the patient positioned in dorsal decubitus [7].

Although ventrodorsal projection was performed in all patients, only the joints of the adult dogs were evaluated and classified according to the OFA grading. The radiographic images were evaluated by three independent radiologists with extensive experience in the interpretation of hip dysplasia, who were blinded to the patient’s history. The joints were classified individually according to the OFA grade: excellent (1), good (2), fair (3), borderline (4), mild (5), moderate (6), and severe (7). Joints were classified as non-dysplastic when two or three raters agreed to classify the joint as non-dysplastic (excellent, good, fair), or dysplastic when they agreed with the disease classification (mild, moderate or severe). Joints classified as borderline or without agreement between non-dysplastic and dysplastic by at least two raters were excluded from the statistical analysis.

Distraction and compression radiographic projections were evaluated by a PennHip-certified veterinarian. The DI was measured as described by Smith et al. [32] with 0 representing full congruence of the hip joint and 1 representing complete dislocation. Animals with a distraction index ≤ 0.5 were classified as non-dysplastic, as according to Flückiger et al. [33]. This is a critical value correlated with the appearance of radiographic signs of degenerative joint disease secondary to hip dysplasia.

After radiographic examinations, all patients were referred for ultrasound examination.

### Ultrasonographic evaluation

All ultrasound examinations were performed by a veterinary sonographer with experience in musculoskeletal evaluations. Trichotomy of the lateral and medial regions of both hip joints to facilitate ultrasound examination of the articular and periarticular structures. Initially, the patients were positioned in lateral decubitus, with the limb to be assessed facing upward, in order to maintain the patient’s comfort and neutral position. For all the techniques performed, ACUSSON S2000™ equipment (Siemens®, Munich, Germany) was used, with a linear transducer and a frequency ranging from 8 to 10Mhz. Ultrasound conductive gel was used throughout the examination. All groups underwent ultrasound evaluation of both hip joints. After the ultrasound examination all dogs were discharged.

### B-mode ultrasound

The structures evaluated were the following muscles: gluteus medius, vastus lateralis, pectineus, gracilis, adductor, and rectus femoris. These muscles were measured according to the evaluation protocol suggested by Carneiro et al. [34]. The thickness of the joint capsule and the bone surfaces of the femoral head and acetabular cranial rim were also evaluated. During the examination, the focus, gain, and depth were adjusted as needed. The characteristics of echogenicity (hypoechogenic or hyperechogenic) and echotexture (homogeneous or heterogeneous) of the muscles were evaluated.

To evaluate the gluteus medius, the transducer was positioned in the craniocaudal direction in the middle third between the greater trochanter and iliac crest, identifying the muscle fibers along its longitudinal axis. The transducer was positioned at 90° relative to the fibers for cross-sectional scanning. The muscle was evaluated along its entire longitudinal and cross-sectional extension.

After identifying the gluteus medius, the transducer was moved distally and caudally to the proximal portion of the thigh (greater trochanter) to identify the vastus muscle which is characterized by its triangular shape. The transducer was then kept in the craniocaudal direction to evaluate the muscle in cross-section. For longitudinal evaluation, the transducer was rotated proximodistally. Due to its distal convergence with other muscles that make up the quadriceps femoris, it was impossible to individualize the vastus lateralis insertion point.

To evaluate the pectineus muscle, the transducer was positioned transversely 90° from the muscle and proximal to the inner part of the thigh. The femoral artery was identified cranial to the muscle and served as a guide for the evaluation. The transducer was then rotated proximodistally to evaluate the longitudinal aspect of the muscles. The muscle was evaluated along its entire longitudinal and cross-sectional extension.

The gracilis muscle was evaluated after pectineus muscle identification. The transducer was moved distally and caudally in the craniocaudal orientation for cross-sectional evaluation. The proximal caudal branch of the femoral artery and vein was evaluated using the Doppler mode and served as a reference point. Subsequently, the transducer was rotated proximal-distal to evaluate the structure longitudinally. The transducer was then moved slightly cranially to assess the adductor. The muscle was then evaluated in the longitudinal and transverse planes.

The pectineus muscle was used as a reference to identify the rectus femoris. Upon identification of the pectineus, the transducer was moved cranially to the limb. The rectus femoris muscle is bipennate, allowing it to be differentiated from other muscles. For transverse evaluation, the transducer was held at 90° relative to the limb and rotated proximally and distally for longitudinal evaluation. Due to its distal convergence with other muscles, it was not possible to individualize this muscle in its insertion. The rectus femoris was assessed proximally and in its middle third.

Hip joint inspection was performed with the transducer positioned dorsally and medially to the greater trochanter in a craniocaudal direction with a dorsoventral rotation of approximately 20° clockwise to the joint. Irregularities on the surface of the femoral head and acetabulum were accounted for; animals with any variations identified in the ultrasonographic inspection were considered dysplastic, and animals that did not have alterations were considered non-dysplastic in the B mode ultrasound evaluation. The articular capsule was measured according to its thickness (height) for comparison with OFA ratings in adult animals and the DI in puppies.

### ARFI elastography

**Fig. 4:**
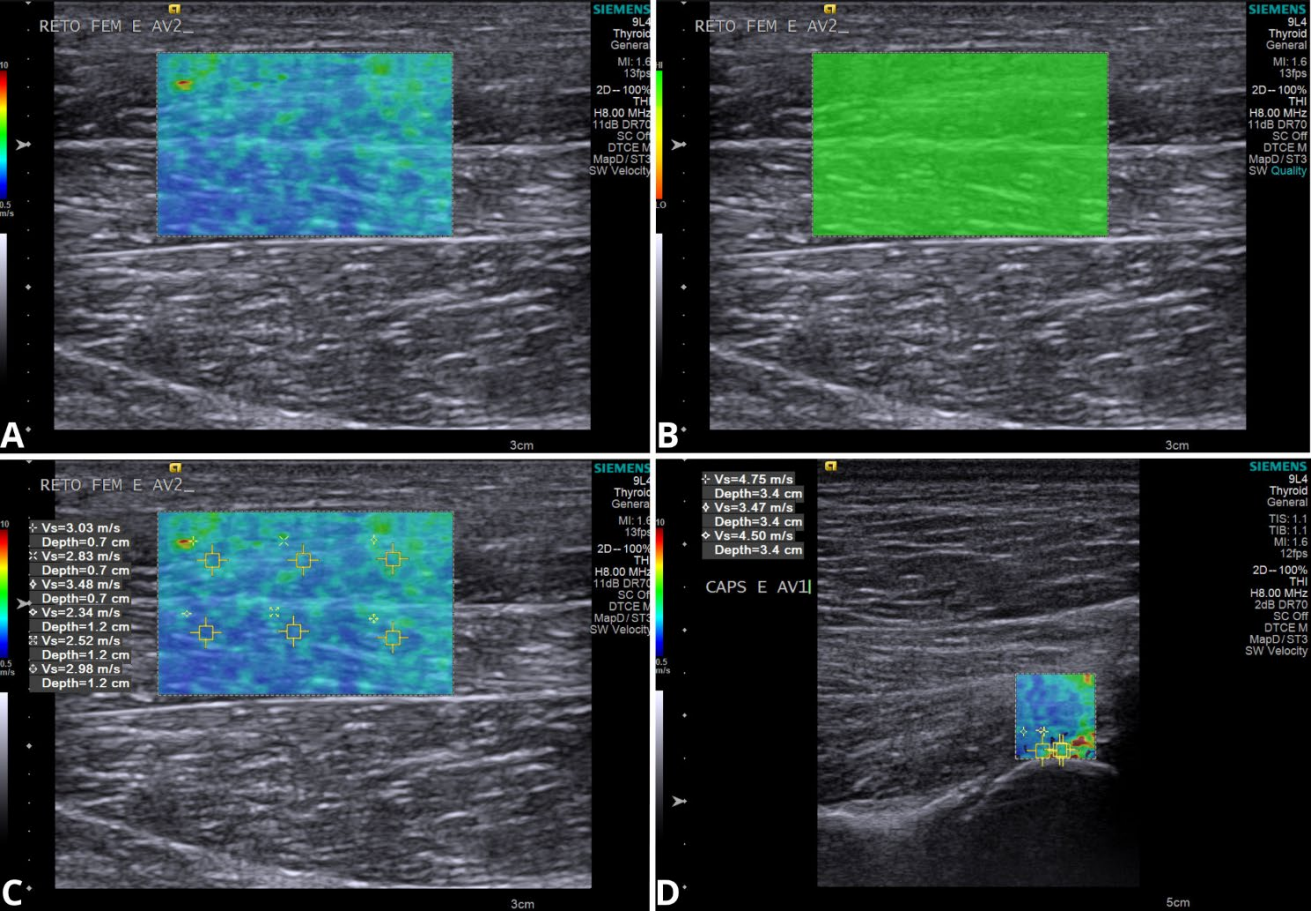
Ultrasonographic images of ARFI elastography in the rectus femoris muscle (**a**, **b** and **c**) and joint capsule (**d**) of the hip joint of a dog. A qualitative study of the aforementioned structures (**a**) demonstrates an elastogram in color, where cold colors represent more elastic areas and warm colors more rigid areas. Quality assessment of the elastogram (**b**) indicates high quality due to the homogeneity of the greenish color. In (**c**) the elastogram of the rectus femoris muscle, the longitudinal section, shows a predominance of blue (slight stiffness), with an average shear wave velocity (SWV) of 2.86 m/s. The (**d**) elastogram of the joint capsule shows a predominance of yellow and green colors (intermediate stiffness) and an average SWV of 4.24 m/s

Elastographic evaluation was performed using the virtual touch tissue imaging quantification, (2D-SWE) technique. In the qualitative study, color elastograms were obtained, wherein the blue colors represent more elastic areas, the green and yellow ones represent intermediate stiffness, and the red ones correspond to the most rigid areas (Fig. 4). Additionally, the quality of the exam was evaluated using the device itself, in which homogeneous and greenish images indicated high quality of the technique, while heterogeneous and yellowish images indicated low quality (Fig. 4). When low-quality images were obtained, the examination was repeated. For quantitative analysis, the same elastograms were used and selected regions of interest (ROI’s) were chosen at random to obtain the average shear wave velocity (SWV – m/s), quantified by the VTIQ software, the value being representative of the total stiffness [23]. Nine ROI’s were selected to assess the gluteal and adductor muscle: six for the vastus lateralis, pectineus, gracilis, and rectus femoris, and three for the joint capsule (Fig. 4). The ARFI Elastographic was used in the central region of the muscle to avoid areas of convergence of muscle fascicles, epimysium, and tendinous regions.

### Statistical analysis

Statistical analyses were performed using the R software version 3.3.0 (R® Foundation for Computational Statistics, Austria). Before carrying out the tests, the mathematical assumptions of normal distribution (Shapiro-Wilk test) and homoscedasticity of variances (Bartlett test) were verified for all collected variables. Real or transformed measurements resulting from ultrasound examination were compared between dysplastic and non-dysplastic patients, classified by DI tests for young group and by OFA test for adult groups, using the Student’s t-test. Subsequently, ultrasound variables that showed significance between dysplastic or non-dysplastic classification were submitted to a diagnostic accuracy test for dysplasia through receiver operating characteristic curve (ROC) analysis in a logistic regression model, calculating the cut-off point, sensitivity, specificity, likelihood ratio, and AUC when the diagnostic accuracy was significant. Finally, trying to identify prognostic or severity factors the studied variables were correlated with each other by Spearman’s test. Data are presented as the mean ± standard deviation (SD), and the significance level was fixed for all tests at P < 0.050.

### Electronic supplementary material

Below is the link to the electronic supplementary material.

Supplementary Material 1

Supplementary Material 2

## Data Availability

All data generated or analysed during this study are included in this published article and its supplementary information files.
